# Evidence for some antimicrobial properties of English churchyard lichens

**DOI:** 10.1099/acmi.0.000536.v4

**Published:** 2023-06-20

**Authors:** J. A. Taylor, Toscane Fourie, Mark Powell, Iva Chianella

**Affiliations:** ^1^​ E.E.E.S and The Graduate School, The Open University, Walton Hall, Milton Keynes MK 7 6AA, UK; ^2^​ INSERM Aix-Marseille University, Provence-Alpes-Côte d'Azur, Marseille, France; ^3^​ British Lichen Society, UK; ^4^​ Cranfield University, Bedfordshire MK43, UK

**Keywords:** lichens, antimicrobial, secondary metabolites, *Diploicia canescens*, *Pertusaria amara*

## Abstract

The emergence of multidrug-resistant bacteria has driven the need for novel antibiotics. Our investigations have focussed on lichens as they naturally produce a wide range of unique and very effective defence chemicals. The aim of this study was to evaluate some of the antimicrobial properties of ten common British churchyard lichens. The lichen material was sampled from ten species, namely *Caloplaca flavescens*, *Diploicia canescens*, *Cladonia fimbriata*, *Psilolechia lucida*, *Lecanora campestris* subsp. *Campestris*, *Lecanora sulphurea, Pertusaria amara f.amara*, *Lepraria incana*, *Porpidia tuberculosa* and *Xanthoria calcicola*. Crude acetone extracts of these lichens were tested against six bacteria (*Escherichia coli, Pseudomonas aeruginosa, Staphylococcus aureus, Salmonela typhimurium, Listeria monocytogenes* and *Lactobacillus acidophilus *) and two fungi (*Trichophyton interdigitale* and *Aspergillus flavus*) by the disc-diffusion susceptibility test method. Extracts of *Diploicia canescens, Psilolechia lucida, Lecanora sulphurea, Pertusaria amara* and *Lepraria incana* showed clear inhibition of the Gram-positive bacteria tested (*S. aureus, L. monocytogenes, L. plantarum*). *Diploicia canescens, Pertusaria amara* and *Lepraria incana* extracts also inhibited the dermatophyte fungi tested. The *Lepraria incana* sample tested here was the only extract that showed activity against any of the Gram-negative bacteria tested; it showed inhibition of *Pseudomnas aeruginosa*. Overall, our results showed that crude extracts of *Diploicia canescens* and *Pertusaria amara* had the most potent antimicrobial activity of all the extracts tested. Our results are in general agreement with published findings elsewhere. The activity of the *Porpidia tuberculosa* margin sample being different from that of the main colony material was an interesting and new finding reported here for the first time.

## Data Summary

Figures and data can be found at at http://doi.org/10.6084/m9.figshare.21689321 [1].

## Introduction

According to the British Lichen Society, over 46 % of British lichen species are found in churchyards [2]. Moreover, church rocks, memorials and boundary walls often have different geological origins within the same churchyard (e.g. limestone, sandstone, ironstone, marble and granite), providing multiple substrates in which specialized lichen communities may develop. Rough, smooth, horizontal and vertical substrates exist in cemeteries, some in shade or in light, some with metal-enriched run-offs. The great variety of microhabitats, shelter a rich diversity of lichen species and they provide, without doubt, sanctuaries for lichens. For this reason, a churchyard was chosen as an appropriate sampling site for our research.

The church in our study was built of local Oolitic limestone during the first half of the fourteenth century and is surrounded by 0.6 ha of cemetery, including gravestones and memorials, some dating from the second half of the seventeenth century but most from 1841 and onwards.

During the Mapping Scheme survey conducted by the British Lichen Society in 1994, 68 species of lichens were recorded. Then, 9 years later, a second survey was conducted by Mr Mark Powell, over 100 species were recorded (M. Powell, personal communication).

Living in extreme conditions and in low-resource habitats, it is not surprising that lichens grow only a few millimetres per year. The authors of [3] have suggested that specific microbiomes are associated with lichens (independent of geography), suggesting perhaps that these microbial assemblages are either prime environments for lichen colonization or protect lichen tissues from predation. The authors of [4] note that ‘There is no evidence that herbivores, pathogens or competitors potentially affected by these compounds are capable of eliminating palatable or poorly defended lichens from communities. Nor is there evidence that species from late successional stages are better defended than pioneers’. However, it has been noted that slow-growing organisms dwelling in low-resource habitats tend to produce higher levels of defence chemicals, likely to ensure their survival [5].

This slow biomass production makes lichens particularly vulnerable to consumers. Historical research has shown that lichens have widely developed chemical strategies to ensure their survival: over 800 secondary metabolites^1^ have been identified [6]. These ‘secondary metabolites’ may protect from parasites, consumers, decomposers and competitors thanks to the large spectrum of antimicrobial, antiherbivory compounds and allelochemicals^2^ [7]. They also protect from environmental stress, such as UV radiation, extreme temperatures and desiccation. These biochemicals are unique to lichen and rarely occur in non-lichenized fungi or higher plants [8]. Secondary metabolites can be used to identify species thanks to chemical testing and taxonomists have reclassified lichen species according to their morphological structures and chemical signatures.

Our study re-visits the antimicrobial activities of churchyard lichens in the light of a perceived need for novel therapeutics in response to the emergence of antimicrobial resistance [9].

Lichens have been used for many centuries as medical remedies but have fallen out of favour with the discovery of chemically synthesized and modified antibiotics in the early twentieth century. However, new analytical methods have helped to identify and isolate many new lichen substances and to test their medical effects. A great variety of effects have been referenced such as antibiotic, antifungal, antiviral, anti-inflammatory, analgesic, antipyretic, antiproliferative and cytotoxic [10]. With the development of methods for cultivating lichen tissue, lichens have now become a potentially interesting source of antibiotics and anticancer drugs [11, 12].

Secondary metabolites are considered to play an ecological role and are usually complex organic acids. Unlike primary metabolites, they are (theoretically) only produced by the fungus, deposited externally (extracellular) and are usually insoluble in water. They are incredibly chemically diverse. Different studies have shown the number to range from 700 [8] to 1050 [13] and most are unique to lichens: they do not occur or rarely in other fungi or higher plants. Only 10 % (approx.) of secondary metabolites have been found in free-living fungi or in the plant kingdom [8]. Interestingly, even if metabolized by the fungal partner only, lichenization is essential to produce these unique secondary metabolites. Indeed, studies have shown that the supply of carbohydrates by the photobiont activates the production of secondary metabolites in the mycobiont [14].

The work presented here aimed to evaluate the antimicrobial properties of common English lichens, in a Buckinghamshire churchyard. To this end, ten lichen species were selected, sampled, extracted and their antibiotic activity was determined by susceptibility tests on pathogenic bacteria and fungi.

Based on the review [11], five bacterial pathogens are recurrently used to assess the potency of antimicrobial agents *Escherichia coli, Pseudomonas aeruginosa, Staphylococcus aureus, Klebsiella pneumonia* and *Bacillus subtilis *. Our study tested three of these organisms, *Escherichia coli* (ATCC 11229)*, Pseudomonas aeruginosa* (ATCC 15442), *Staphylococcus aureus* (ATCC 6538)*,* and substituted *Salmonella typhimurium* (ATCC 53648, a Gram-negative rod in place of *Klebsiella pneumonia*) and *Listeria monocytogenes* (ATCC 13932), and *Lactobacillus plantarum* (NCIMB 11974) as substitute Gram-positives in place of *Bacillus subtilis *. The choice of these test organisms was based upon their availability, ease of culture and representation of both Gram-positive and negative bacterial organisms thus potentially showing lichen antimicrobial activities against differing bacterial wall chemistries.

Antifungal properties of lichens are researched for agricultural, food science and medicinal purposes. Each sector has their fungi of interest. Agriculture aimed research focuses on fungi responsible for plant diseases [15], food science research for food spoilage and medicinal research on dermatophytosis [16] or other fungal infections. In this study we tested *Trichophyton interdigitale* (clinical isolate 4167167) and *Aspergillus flavus* (ATCC 200026, also referred to as NRRL 3357) as these are medically significant and are responsible for nail infections and aspergillosis.

## Methods

### Positive and negative controls

The negative control in this study consisted of a sample made from completion of a Soxhlet extraction procedure in the absence of lichen material. The positive control ensured that the test micro-organisms were not resistant to drugs. Generally, a broad-spectrum antibiotic is used: usually ketoconazole for fungi [17–21] and chloramphenicol for the bacteria tested in these experiments. Results were expected to be positive (i.e. with antimicrobial effects) for both positive controls. The inhibition zones produced by the positive controls were compared to those produced by the test samples and indicated the strength of lichen antimicrobial activity.

### Sampling lichen material

Lichen material was obtained from the churchyard, which benefits from an oceanic climate similar to the rest of the Midlands. On average, rainfall is annually constant with 10 days of rainfall≥1 mm per month throughout the year. The temperatures are annually cool, varying on an average from 6.36 °C to 14.37 °C [22]. Permission to conduct this study and collect materials was given by the Churchwarden and the PCC.

The selection of ten species was based on the three following criteria:

1. Species where the genus showed some evidence of antimicrobial activity within the literature.
2. On-site observations suggesting competition between lichens.
3. Sufficient abundance on the site.

The samples were collected from colonies identified by the expert (M. Powell). With a knife or sand paper for very thin and embedded lichens, the material was scraped from its stone substrate on to the foil. The sample was weighed on site. The colony margin sample of prothallus material (sample 8) was collected carefully by only scraping the dark boundary material of the colony and avoiding the surrounding adjacent lichens as much as was possible.

### Lichen species selected for study

Sample 1: *Caloplaca flavescens* (Huds.) J.R. Laundon

Sample 2: *Diploicia canescens* (Dicks.) A. Massal.

Sample 3: *Cladonia fimbriata* (L.) Fr.

Sample 4: *Psilolechia lucida* (Ach.) M. Choisy

Sample 5: Lecanora *campestris* (Schaer.) Hue subsp. Campestris

Sample 6: Pertusaria amara f. amara (Ach.) Nyl.

Sample 7: *Lecanora sulphurea* (Hoffm.) Ach.

Samples 8 & 10: *Porpidia tuberculosa* (Sm.) Hertel & Knoph

(one sample from main colony thallus and one from colony margin)

Sample 9: *Lepraria incana* (L.) Ach.

Sample 11: Xanthoria calcicola Oxner

### Lichen extracts’ extraction of the secondary metabolites

Lichen extracts were generated by the Soxhlet extraction method. Air-dried samples were ground with a mortar and a pestle and resultant powders were successively extracted in Soxhlet apparatus (Quickfit, 34/35 diameter) folded in filter paper (Whatman grade 3 qualitative filter paper, pore size: 6 µm, Merck WHA1003240) for 7.5 hours to 8 hours. 270 ml of acetone (Sigma-Aldrich, 179124) was used as the solvent for the extraction. The method was based on principles employed in [23].

After extraction, the acetone extract was collected into a beaker and left in the fume cupboard at room temperature to evaporate. Once the solvent evaporated, the extracted chemical extracts were evident in the bases of their respective beakers. Crystals were observed in all beakers. The dried extracts were stored at room temperature, the beaker sealed with parafilm (Sigma-Aldrich, P7793) to avoid physical contamination.

### Preparation of extracts’ liquid solutions

Liquid solutions were prepared with acetone (Sigma-Aldrich, 179124) to impregnate small paper discs for the antibiotic susceptibility testing.

According to the observations of [24], it is advantageous to use the same solvent for suspension of the dried crystalline extracts as was used for the extraction process. This advice was followed in later studies [25, 26]. Therefore in this study, acetone was used to dissolve the extracts.

### Concentrated solutions

The lichen extract solutions were adjusted to be of comparable concentrations (as far as possible within the constraints of the variability of the lichen sample sizes available) such that the paper discs impregnated with them would yield comparable data. In the literature, an average quantity of crude extract per disc was 300 µg, made from a stock solution containing an extract concentration of 30 mg ml^−1^ [21, 25]. To emulate this, our dried extracts were dissolved in acetone (Sigma-Aldrich, 179124) to a final theoretical concentration of 60 mg ml^−1^ or 30 mg ml^−1^. The details of calculations on which these dilutions were based are shown in Table 1. The solutions were stored at 4 °C until required for assay experiments.

**Table 1.** Preparation of stock series of lichen extract solutions. Dilutions made in acetone (Sigma-Aldrich, 179124) according to methods described above to generate final solutions of either c.30 mg ml^−1^ of 60 mg ml^−1^

Sample	wt of dry extract (mg)	Acetone vol. added (ml)	concn factor from S1 to S2	Final theoretical concn S2 (mg ml^−1^)
**1**	17	0.7	85.7	24.29
**2**	70	1.16	43.1	60.34
**3**	30	1	40	30
**4**	50	0.83	30.1	60.24
**5**	40	1.33	45.1	30.08
**6**	150	2.5	24	60
**7**	170	2.83	21.2	60.07
**8**	3	0.4	27.5	7.5
**9**	170	2.83	21.2	60.07
**10**	230	3.83	14.4	60.05
**11**	100	1.67	35.9	59.88

**Key**

**S1** Original theoretical concentration of lichen extract solution.

**S2** Final theoretical concentration of lichen extract solution.

### Calculation of extraction yields

The extraction yield is the percentage (by weight) of lichen material extracted during the 8 h Soxhlet extraction with acetone solvent:

$$Extraction\;yield=\frac{\;C_{s1}\;\times V_{sample}}{m_{sample}}$$

with *V*_sample_ the volume of acetone (in ml) added to dissolve dried extract into *S*_1_ (cf. values in Table 1); *m*_sample_ the mass of the lichen sample (in mg) used for the extraction; *C_s_*_1_ the concentration of extract in *S*_1_ (in mg ml^−1^).

Example for sample 1:

$$Extraction\;yield\;1=\frac{\;C_{s1}\;\times V_{sample1}}{m_{sample1}}=\frac{0.2833\;\times 100}{3000}=0.00944=0.94\%$$

### Antibiotic susceptibility assay

The antibiotic susceptibility assay involved three steps detailed in the following paragraphs.

### Preparation of susceptibility testing discs

Under laminar flow conditions, multiple applications of 10 µl aliquots of solutions were added to sterile filter discs (blank antimicrobial susceptibility test discs, Oxoid, diameter: 6 mm, WHA2017006) allowing evaporating between applications (two applications of 5 µl each). Once dried, the discs were stored in screw vials at 4 °C until susceptibility testing. The disc loads (µg) for each sample tested are shown in Table 2.

**Table 2.** Load of lichen extract (µg) per Whatman test disc for each lichen extract sample (extracts prepared according to the methods described above)

Sample	1	2	3	4	5	6	7	8	9	10	11
Final theoretical concentration of S2 (mg ml^−1^)	24.3	60.3	30	60.2	30	60	60	7.5	60	60	59.8
Volume per discs (µl)	10	10	10	10	10	10	10	10	10	10	10
Load per discs (µg)	243	603	300	602	300	600	600	75	600	600	598

The negative control disc’s role underlines the potential effects of acetone as well as aluminium salt and grease used in the Soxhlet extraction. The solution used to impregnate the negative control disc was prepared by running a Soxhlet extraction without a sample in the extraction chamber.

The positive antibacterial agent selected was chloramphenicol (chloramphenicol Fisher BioReagents, Fisher Scientific, Cat. No. 10255203) and the antifungal agent was Prochloraz (Merck, UK). Antibacterial positive control discs were impregnated with 10 µl of a 2.5 mg ml^−1^ of chloramphenicol acetone solution (determined by experimentation, data not shown). Antifungal positive control discs were impregnated with 30 µl of a 45 ppm (45 mg l^−1^) Prochloraz solution (determined by experimentation, data not shown).

### Preparation of agar plates and inocula

Test organisms were grown on a range of selective and non-selective media according to their nutritional requirements and according to cost-effective availability. The media chosen were as follows (agar and broth): Tryptone Soy medium for S.*aureus*, *P.aeruginosa* and *S.typhimurium* (Oxoid CM0129 and CM0131 supplied by ThermoFisher Scientific, CM0129B and CM0131B); MRS medium for *L. plantarum* (Oxoid CM0359 and CM0361 supplied by ThermoFisher Scientific, CM0359B and CM0361B); Listeria selective agar (Oxford formulation) for *L. monocytogenes* (Oxoid CM0856 supplied by ThermoFisher Scientific, CM0856B); and Sabouraud dextrose agar for fungi (Oxoid CM0041 supplied by ThermoFisher Scientific, CM00041R). Medium solutions were prepared with distilled water and were autoclaved as per the manufacturer's recommended methods. Media were poured (aseptic techniques) into sterile Petri dishes (Sterilin, diameter: 90 mm supplied by ThermoFisher Scientific, 15370366) under laminar flow conditions. Once the media solidified, the Petri dishes were stored at room temperature. Unlike refrigerated storage, room-temperature storage enables an easy identification of contaminated plates.

Pure bacterial strains were inoculated into sterile Erlenmeyer flasks containing 100 ml of adequate broth, under laminar flow conditions. The broth was incubated overnight at 37 °C. After an overnight incubation, broth should contain 10^8^ cells ml^−1^. This number was confirmed by a direct microscopic count of *E. coli* and *L. monocytogenes* overnight broths using a THOMA haemocytometer.

Using aseptic techniques, spores from pure fungal colonies were collected into sterile Tween water in a centrifuge tube. The suspension was centrifuged for 5 min at 1000 r.p.m. The supernatant was discarded; the spores were suspended in 10 ml of sterile water for spore counting in the THOMA haemocytometer. Centrifuged a second time for 5 min, the supernatant was discarded and, according to the spore count, the spores were suspended into the appropriate volume of sterile water to achieve an ideal concentration of 10^6^ spores ml^−1^.

### Incubation procedure

All experimental plate (microbe and lichen-impregnated disc) combinations were prepared in triplicate. Using aseptic techniques, 100 µl of spore inoculum or bacterial O/N culture was used to inoculate the appropriate plates. The use of a glass spreader helped to ensure uniformity of the microbial lawns. Impregnated discs were applied to the plate within 15 min of inoculation. Plates incubated 24 h at 37 °C for bacteria, except *Lactobacillus plantarum,* which incubated at 30 °C, and 72 h at 30 °C for fungi.

## Results

### Extraction-yield results

The extraction yields are detailed in Table 3. Extraction yields varied from 0.9–13.9 %. A wide range of yields among the extracts was observed. *Caloplaca flavescens* (sample 1) and *Porpidia tuberculosa*’s margin (sample 8) exhibited the lowest extract yield, while *Porpidia tuberculosa*’s thallus (sample 10) exhibited the highest extraction yield.

**Table 3.** Percentage extraction yields of acetone lichen extract samples prepared in this study according to the methods described above

Sample ID	Mass of sample (mg)	vol. of S1 evaporated (ml)	concn of dry residue in S1 (mg ml^−1^)	% extraction yield
	***m***	***Vs1***	***Cs1***	
**1**	3000	60	0.283333	0.94
**2**	3000	50	1.4	4.66
**3**	3000	40	0.75	2.50
**4**	1000	25	2.0	6.67
**5**	3000	60	0.66	2.22
**6**	3000	60	2.5	8.33
**7**	2000	60	2.83	9.44
**8**	500	11	0.272	0.91
**9**	3000	60	2.83	9.44
**10**	2000	55	4.18	13.94
**11**	3000	60	1.66	5.55

The results of screening the lichen acetone extracts for antibacterial and antifungal activity are presented in Table 4.

**Table 4.** Mean Inhibition zone measurements (diameter in mm) of lichen extract susceptibility tests on Gram-positive and Gram-negative bacterial and fungal culture growth KEY: All experiments completed in triplicate for each bacterial/fungal culture tested (mean and standard deviation shown). “Sample” denotes lichen extract tested. * denotes colony thallus margin sample. Load in µg is amount of lichen extract impregnated into test disc (+) denotes positive control disc experiment (disc impregnated with Prochloraz solution (fungal experiments) and with chloramphenicol (bacterial experiments) (-) denotes negative control disc experiments (disc impregnated with acetone from control Soxhlet procedure) 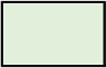
 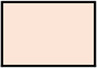
no inhibition/sensitivity zone observed. (NT denotes not tested due to insufficient material, C denotes contamination).

Lichen extract		Gram-negative bacteria						Gram-positive bacteria						Fungi			
		* E. coli *		* P. aeruginosa *		* S. typhimurium *		* S. aureus *		* L. monocytogenes *		* L. plantarum *		*T. interdigitale*		*A. flavus*	
Sample	Load μg	mean	sd	mean	sd	mean	sd	mean	sd	mean	sd	mean	sd	mean	sd	mean	sd
1. *C.flavescens*	243																
2. *D.canescens*	603							14	0.6	13.8	0.3	7.5	0	13	1.2		
3. *C.fimbriata*	300													7.2	0.3		
4. *P. lucida*	602							9.8	0.6	8.3	2.3						
5. *L. campestris*	300													C			
6. *P. amara*	600							14	0.6	12.5	0.5	14	0.3	11.7	3.2		
7. *L. sulphurea*	600							9.5	1	8.5	4.9	8.5	0.5	7.3	4.2		
8. *P. tuberculosa*	75							8.3	0.6	9.3	1.2	nt		nt		nt	
9. *L. incana*	600			9	1			7.2	0.3	8	0	8.2	0.3	8.8	0.3		
10. *P. tuberculosa*	600													8.5	4.9		
11. *X. calcicola*	598																
(+) control	25	23	0	14	8	18	1.2	21	0.6	17.3	0.6	21	3	13.7	1.5	22.3	2.9
(-) control	1.35																

Table 4 shows that six extracts out of eleven tested showed activity against Gram-positive bacteria, diameters of inhibition zones varying from 7 to 14 mm. The extract of *Pertusaria amara* showed the most potent antibacterial activity with inhibition zones between 13 and 14 mm.

It is also important to note that the extract of *Porpidia tuberculosa*’s margin (sample 8) inhibited the growth of Gram-positive bacteria tested, whereas *Porpidia tuberculosa*’s main thallus tissue sample (sample 10) did not. *Staphylococcus aureus* and *Listeria monocytogenes* were sensitive to smaller doses of extract from *Porpidia tuberculosa*’s margins when they were resistant to an extract from its thallus.

The lichen extract doses tested here, were generally ineffective at controlling the growth of the Gram-negative bacteria tested. Although a very weak activity was identified for *Lepraria incana* against *Pseudomonas aeruginosa *. These results indicated that the *Lepraria incana* extract inhibited *Pseudomonas aeruginosa *, a bacterium naturally resistant to a large spectrum of antibiotics, including chloramphenicol.

Table 4 also shows that only the dermatophytic fungi (*T. interdigitale*) was inhibited by the lichen extracts. *A. flavus* was not inhibited by any of the extracts tested.

*T. interdigitale* showed most sensitivity to *Diploica canescens* (the most potent antifungal activity seen in these experiments with inhibition zones measuring up to 14 mm), but also to lesser degrees to *Pertusaria amara* and *Lecanora incana. T. interdigitale* also showed a lesser sensitivity t*o Cladonia fimbriata, Lecanora Sulphurea* and *Porpidia tuberculosa* extracts.

## Discussion

Deficient infection and disease control, limited access to clean water, poor human/animal sanitation and hygiene, in both animal and human care environments and the misuse of antimicrobials will continue to increase the spread of antimicrobial resistance genes. This situation will increase the rate of emergence of new resistant bacterial pathogens and thus heighten the requirement for new, efficacious antimicrobial agents [9, 27, 28].

Historically lichens have been reported to exhibit antimicrobial activity both within the context of science and folklore [29]. It may be time to re-evaluate these overlooked antimicrobial sources as requirements for novel treatments grow.

To this end, the antimicrobial activity of crude acetone extracts of ten common English churchyard lichens were tested by a disc-diffusion susceptibility test against five pathogenic bacteria, a probiotic bacterium, an anthropophilic dermatophyte fungus and a mycotoxin-producing fungus.

The concept of ‘warfare-like’ competition between adjacent lichen colonies was alluded to when Pentecost [30], first coined the term ‘truce’ when observing the habit of expanding lichen colonies as they grew and abutted at their colonial edges. Competing lichens were deemed ‘well matched’ at a site where no colony overgrowth of abutting colonies and no overall ‘outcompeting winner’ could be declared. Pentecost’s work obviously related to interaction between colonies, the perception of boundaries and the sensing of the presence via physical sensation or chemical detection. Dale [31] discussed inter and intraspecific competition between crustose lichens and again described the ‘truce boundary’, but as an indication of growth rates. Interactions such as these two papers observed, based on a chemical interaction and perception at hyphal tips, is the basis of our research paper and these ideas informed our experiments.

Seven of the ten lichen samples tested in this study exhibited some activity against bacteria and/or fungi. Gram-positive bacteria were affected more than Gram-negative bacteria but only the dermatophytic fungi showed sensitivity to lichen extract.

The most potent lichen extracts tested in the experiments within the research presented here were *Diploicia canescens* and *Perturaria amara*. Both are known for producing diploicin and picrolichenic acid, respectively, as major metabolites. *Diploicia canescens* is also rich in atranorin and numerous chlorinated depsidones and depsides with cytotoxic effects [32]. Both of these lichen extracts were active against *S. aureus *, the most pathogenic species of the genus of which disease-related strains produce exotoxins and are the most common post-surgical nosocomial infections. The hospital environment has created natural selection for antibiotic-resistant microbes, including methicillin-resistant *Staphylococcus aureus* (MRSA) strains, which are hardest to treat.

Diploicin was first isolated by Kopf in 1904 from *Diploicia canescens* (named *Buellia canescens* at the time) and has since been found to be very active against *Mycobacterim tuberculosis* [33]. Atranorin (also extracted from other lichens) has proved to be effective against Gram-positive bacteria [34] and in particular against MRSA [35]. *Pertusaria amara* was first identified by Alms in 1832 and named by Kopf in 1900. Interestingly, no online documentation has been found concerning its antibiotic activity or that of its major metabolite, picrolichenic acid.

The activities of *Diploicia canescens* and *Pertusaria.amara* extracts against *T. interdigitale* were the strongest reactions shown in this study. The activity shown by *Diploicia canescens* being equivalent to that of the positive control agent. *Diploicia canescens* is susceptible to attack by licheniculous fungi and as such, perhaps, antifungal activity may be a variable within populations of this organism. The work of [36] suggested that both altitude and light levels can affect the concentrations of secondary metabolites produced within a species. Alternatively, the strains of *Diploicia canescens* tested in this study may be resistant to licheniculous fungi, and the anti-fungal activity observed here could be the result of such chemical resistance.

Antimicrobial activity was also observed in this study in relation to samples of *Porpidia tuberculosa*, but results indicated that antimicrobial compounds were unevenly distributed within the thallus of the sampled material. The extract from the periphery of the thallus showed strong activity against Gram-positive organisms. Results suggest that the accumulation of defence compounds in *Porpidia tuberculosa’s* margins could be an ecological strategy to aid substrate colonization or prevent fungal attack [37]. Interesting on-site observations led to the selection of this species. Indeed, the margin of the lichen was delimited by a narrow black-brown prothallus. *Porpidia tuberculosa* was in competition for substrate coverage and was seemingly ‘conquering’ the surface over other species of lichens that were present. A sample was collected that contained mainly *Porpidia tuberculosa*’s thallus and a second contained the prothallus and margin with other lichen species (mainly *Lecanora sulphurea* because a sample comprising exclusively of margin was impossible to collect). These two samples allowed us to test whether prothallus margins contain more allelopathic/antimicrobial compounds than the centre of the colony thallus. Our experiments permitted us to answer this question in so far as that the potential contaminant *Lecanora sulphurea* (which grew adjacent to *Porpidia* at the sampling site) showed less activity against *L. monocytogenes* than the *Porpidia* margin sample, which was eight times less concentrated. The activity of the *Lecanora sulphurea* extract against *S.aureus* was similar in intensity to that of the *Porpidia tuberculosa* margin extract, but it is important to remember that this margin sample was at a lower concentration than other extracts tested. By proportion then, it could be suggested that the margin sample was more potent than other extracts tested in this study and that margin tissue does truly have a higher concentration of antimicrobial agents than central thallus tissue [38]. It was unfortunate that there was insufficient margin material to test against the fungal species in this study, although encouraging that *Porpidia tuberculosa* inhibited antimicrobial activity against *T. interdigiale*. The authors of [39] presented evidence that both usnic acid and perlatonic acids, not being water soluble, do not accumulate in soils underneath lichens, in fact the highest concentrations are found only at apical growing tips of lichens. This theory would hold with our tentative findings regarding increased antimicrobial activity being associated with prothallus ‘black line’ material collected from *Porpidia tuberculosa*’s margin.

Even though *Caloplaca flavescens* is very common in the British Isles, its metabolites have not been identified. Literature for the genus *Caloplaca*, show the presence of anthraquinones with broad-spectrum biological activities, including antifungal and antibacterial properties [40]. No inhibitory activity was seen in this study with any of the bacteria tested.

*Cladonia fimbriata* has proven antibiotic properties [21]. Rankovic’s study showed *Cladonia’s* antimicrobial activity in 5 out of 6 of the bacterial strains tested (with *E. coli* being most resistant) and 5 out of 11 of the fungal strains tested. This activity may be justified by *Cladonia fimbriata*’s major metabolite, fumarprotocetraric acid: a highly active antimicrobial compound [19]. Surprisingly, no inhibitory activity was seen in our study with any of the bacteria tested. This could perhaps suggest that environmental conditions affect the antimicrobial properties of a species [3, 36]. *Psilolechia lucida* contains rhizocarpic acid (a pulvinic acid derivative), used to identify the species from the other *Psilolechia* lichens through a spot-colour test [41]. This metabolite has shown interesting antimicrobial effects against MRSA strains. The activity being comparable to or better than the levels of clinically used antibacterial drugs [42]. In our study, *Psilolechia’s* inhibitory activity was recorded against both *S. aureus* and *L. monocytogenes *.

When *Lecanora* lichens react positively in the potassium spot test (a drop of 10 % KOH applied to thallus) [41], they turn yellow; this is because atranorin (depside) is present, which has proven antimicrobial activity. In the literature, the genus *Leconora* shows broad spectrum antibacterial and some antifungal properties with *Lecanora atra,* and *Lecanora muralis* [43] and *Lecanora frustulosa. Lecanora campestris* contains depside, but our study showed no activity against either bacteria or fungi, again perhaps due to localised environmental conditions.

According to [44], *Lepraria incana* always produces divaricatic acid (depside) but variably produces accessory metabolites such as atranorin (depside), parietin (anthraquinone), zeorin (terpene) and thamnolic acid. When extracted from other lichens, divaricatic acid and zeorin have shown a broad-spectrum antimicrobial activity (inhibition on 5/6 bacteria and 10/10 fungal strains) [21]. In our study, this extract was the only one to suggest inhibition of a Gram-negative organism (* P. aeruginosa *). *P. aeruginosa* is included in the ESKAPE pathogens outlined by [45] and reinforced by [46] in their review.

A Spanish study identified atranorin (depside) in *Xanthoria calcicola*. The study showed little inhibition against Gram-negative *Klebsiella pneumoniae *, but none against *Bacillus cereus *, *Bacillus megaterium *, *Staphylococcus aureus *, *Escherichia coli* and *Pseudomonas aeruginosa* [47]. The inclusion of this species in our study could be considered as a negative control species and indeed showed no activity against either bacteria or fungi.

The authors of [48] note how the main classes of medically significant antibiotics target a limited number of proteins that are involved in bacterial cell-wall biosynthesis, protein synthesis and DNA replication and repair.

Antimicrobial activity, such as observed here, in a single lichen extract sample against both Gram-positive, Gram-negative and fungal organisms, suggests a diversity of active antimicrobial defence, and/or advancement strategies within that lichen. Thus, a lichen may contain multiple agents that can undermine Gram-positive wall structural elements such as peptidoglycan and perhaps other agents that can also interfere with fungal [48] wall chitin synthesis as well as an array of other metabolic processes such as DNA repair [48]. The authors of [49] noted how depsidones have antimicrobial activity against both Gram-positive and negative bacteria and fungi, indeed some are RecA inhibitors which ‘potentiate bactericidal activity and reduce antibiotic resistance’ [48].

The authors of [50] describe how bacteria use the SOS response pathway in response to adversarial challenges including antimicrobial damage. RecA (stress sensor protein) and LexA (stress effector protein) control this response as they are involved in DNA repair and mutagenesis. The SOS response is related to the acquisition of resistance genes. Thus agents that interfere with RecA (depsidones) could interfere with SOS repair and could interfere with the SOS response and reduce the transmission of antimicrobial resistance genes.

*T. interdigitale* showed a range of susceptibilities to the lichen extracts tested in this study. The absence of antimicrobial activity from any of the lichens tested here against *Aspergillus flavus* is however of interest. Perhaps the thick-walled conidia of *A. flavus* protect from adverse environmental conditions such as our lichen extracts [51].

Is indeed any of the antimicrobial activity presented in this study solely attributable to the lichen species selected and sampled, or could it be attributable to bacteria living in close association with these lichen colonies [52]? All our samples were collected from the field and clearly, were thus contaminated with microbes from the adjacent lichen microbiome. We need to be aware that the antimicrobial activities we have attributed to lichen in our experiments, may be associated with either endophytic or endolichenic fungi and/or non-photoautotrophic bacteria living in close association with lichen tissues we sampled. The work of [53, 54] details how lichen-associated bacterial communities provide resistance against biotic stress factors, provide vitamins, nutrients and aid in the ‘recycling’ of degrading lichen tissues. Bacterial associations with algae and fungi may be species-specific rather than geographically significant, i.e. a consortia associated with a lichen rather than a geology or a geography. [3] Sierra’s study of the microbiomes associated with seven lichen genera show a host specificity; ‘These microbiomes varied among lichens and were distinguished based on host identity rather than location or growth substrate’. It is logical therefore to suppose that antimicrobial activity associated with lichens may be attributable to their microbiome as much as to their myco and photobionts. Our results may indeed be attributable to these lichen-associated bacteria.

### Final comments and future work

Seven of the ten lichen samples tested exhibited some activity against bacteria and/or fungi. Gram-positive bacteria were affected more than Gram-negative bacteria but only the dermatophytic fungi showed sensitivity to lichen extract.

Further studies will determine the MIC of the active extracts and isolate their metabolites in order to identify the potential novelty of the agents responsible for antimicrobial activity and whether they are lichen or bacterial in origin.

## Footnotes

Secondary metabolites are products of the metabolism that are not required for the organism to survive. The compounds are not directly involved in growth, development or reproduction.

Allelochemicals are biochemical produces by a plant that influences the survival of another species. The phenomenon is called allelopathy. The term is commonly used for negative allelopathy, in which the plant inhibits the growth of the other.
